# 3-Chloroplumbagin Induces Cell Death in Breast Cancer Cells Through MAPK-Mediated Mcl-1 Inhibition

**DOI:** 10.3389/fphar.2019.00784

**Published:** 2019-07-26

**Authors:** Anna Kawiak, Anna Domachowska, Aleksandra Krolicka, Monika Smolarska, Ewa Lojkowska

**Affiliations:** Department of Biotechnology, Intercollegiate Faculty of Biotechnology UG and MUG, University of Gdansk, Gdansk, Poland

**Keywords:** apoptosis, breast cancer, MAPK pathway, Mcl-1, plumbagin derivatives

## Abstract

Resistance acquired toward anti-cancer agents is a significant drawback in breast cancer therapy. A key factor contributing to drug resistance is apoptosis suppression associated with the upregulation of anti-apoptotic Bcl-2 family proteins. Specifically, the anti-apoptotic Mcl-1 protein has been shown to play a significant role in drug resistance, making it an important therapeutic target. The present study aimed at determining the antiproliferative activity of 3-chloroplumbagin (ChPL), a naphthoquinone derived from a *Dionaea* sp., toward breast cancer cells and examining the involvement of Mcl-1 inhibition in ChPL-induced cell death. The results showed that ChPL inhibited breast cancer cell proliferation and induced apoptosis through the intrinsic pathway through down-regulation of anti-apoptotic Bcl-2 family proteins. The induction of apoptosis by ChPL was found to be mediated through MAP kinase signaling inhibition. ChPL inhibited the phosphorylation of MEK and ERK proteins in breast cancer cells, and increased apoptosis induction in cells with reduced ERK expression. Furthermore, ERK silencing decreased the expression of Mcl-1 in ChPL-treated cells. The results of this research indicate that ChPL induces apoptosis in breast cancer cells through MAPK-mediated Mcl-1 inhibition, suggesting further research into its potential in breast cancer treatment.

## Introduction

Breast cancer is among the most frequently diagnosed cancers in the female population. Despite advances in breast cancer treatment, a major obstacle in treatment remains resistance acquired toward therapy. One of the factors associated with resistance to therapy is the evasion of apoptosis by cancer cells (Hanahan et al., 2000). Specifically, the upregulation of the anti-apoptotic Bcl-2 family proteins has been shown to suppress cell death induced by cytotoxic anticancer drugs (Yip and Reed, 2008). The majority of cytotoxic anticancer agents depend to a large extent on the intrinsic, Bcl-2/Bax-dependent mechanism of cell death induction. This renders the anti-apoptotic Bcl-2 proteins important targets in cancer drug design (Yip and Reed, 2008). The anti-apoptotic Bcl-2 and Bcl-X_L_ proteins have been the main focus of targeted inhibitor design (Vogler et al., 2009). However, recent research has pointed to the importance of targeting the anti-apoptotic Bcl-2 family protein, Mcl-1 (Willis et al., 2005). Mcl-1 overexpression has been reported in breast cancer cells and has been linked with poor prognosis in breast cancer patients and resistance to drug-mediated apoptosis induction (Ding et al., 2007; Booy et al., 2011). The MAP kinase signaling pathway, which plays an important role in breast cancer progression, has been shown to regulate Mcl-1 expression (Mueller et al., 2000). MAP kinase activation increases Mcl-1 expression leading to cell survival, implicating the role of Mcl-1 in apoptotic cancer cell evasion and breast cancer therapeutic resistance (Williams and Cook, 2015).

Plant-derived natural products are an important source of compounds with medicinal potential, often serving as drug leads (Atanasov et al., 2015). 3-Chloroplumbagin (ChPL) is a naphthoquinone present in plants from the Droseraceae and Plumbaginaceae families (Sidhu and Sankaram, 1971; Krychowiak et al., 2014). ChPL is a derivative of plumbagin (PL), a naphthoquinone with anti-proliferative activity toward various cancer cell lines (Kawiak et al., 2007; Aziz et al., 2008; Shieh et al., 2010), including breast cancer (Lee et al., 2012; Ahmad et al., 2008; Manu et al., 2011). Our previous research revealed the ability of plumbagin to induce apoptosis in breast cancer cells and sensitize breast cancer to tamoxifen-induced apoptosis induction (Kawiak et al., 2012a; Kawiak et al., 2017). *In vivo* studies have demonstrated the potential of plumbagin to inhibit tumor growth in mice (Kuo et al., 2006). Plumbagin has been shown to induce apoptosis through the downregulation of the anti-apoptotic Bcl-2 family proteins, and among them, Mcl-1 was found to be downregulated by PL in leukemia cells (Kawiak et al., 2012a; Gaascht et al., 2014). Previous studies have investigated plumbagin as a lead compound in the development of derivatives with higher therapeutic properties (Dandawate et al., 2014). The present research focuses on examining the activity of a 3-chloro derivative of plumbagin and is the first report on the anti-proliferative properties of this compound. The ability of ChPL to induce apoptosis in breast cancer cells was examined and the mechanism of ChPL-induced cell death was investigated.

## Materials and methods

### Plant Material

The source of ChPL were 8-week-old *Dionaea muscipula* plants cultured *in vitro* according to a previously published procedure (Szpitter et al., 2014).

### Isolation of ChPL

The extraction of plant material was performed according to the previously published procedure (Kawiak et al., 2012b). Briefly, dried plant material was sonicated for 30 min in chloroform. Following centrifugation and evaporation, the obtained crude extract was dissolved in chloroform and separated on a silica gel column. Isolation was performed using a step gradient of methylene chloride in hexane. ChPL (PubChem CID: 338719) was obtained as yellow-orange plates, mp 113°C to 115°C, spectroscopic data comparable to literature data (Kreher et al., 1990).

### Chemicals

Materials and chemicals, if not otherwise specified, were purchased from Sigma-Aldrich (St. Louis, MO, USA).

### Cell Culture

The MCF-7 and MDA-MB-468 breast cancer cell lines were purchased from Cell Lines Service (CLS, Germany) and the MCF 10A cell line from the American Type Cell Collection (ATCC, LGC Standards). MCF-7 and MDA-MB-468 cells were cultured in RPMI medium supplemented with 10% fetal bovine serum and 2 mM glutamine. MCF 10A cells were cultured in DMEM/F12 medium supplemented with 5% horse serum, 2 mM glutamine, 20 ng/ml epidermal growth factor, 500 ng/ml hydrocortisone, 100 ng/ml cholera toxin, and 10 μg/ml insulin. All cell cultures also contained 100 units/ml penicillin and 100 mg/ml streptomycin and were maintained in an incubator (Heraceus, HERAcell) in a humidified atmosphere with 5% CO_2_ at 37°C.

### Cytotoxicity Assay

Cell viability was determined using the MTT [(3-(4,5-dimethylthiazol-2-yl)-2,5-diphenyltetrazolium bromide] assay. MDA-MB-468 and MCF7 cells were plated at 5 × 10^3^ cells/well in 96-well plates. Cells were treated with ChPL (0–5 μM) for 24, 48, and 72 h, and with PL (0–5 μM) for 72 h. MCF 10A cells were treated with ChPL and PL for 72 h. Analysis was carried out as previously published (Kawiak et al., 2012b).

### Synergistic Activity Determination

The combined effects of ChPL and paclitaxel (PTX) on breast cancer cell viability were determined with the use of the Chou and Talalay (1984) method. MDA-MB-468 cells were treated with ChPL (μM) and PTX (nM) at the following fixed combinations: 0.1/0.1; 0.2/1; 0.5/5; 1/10; 2/20. The combination index (CI) was calculated as previously published (Kawiak et al., 2019). Obtained CI values lower than 1 indicate synergistic activity between compounds, whereas CI values higher or equal to 1 indicate antagonistic and additive activity, respectively.

### Annexin V Staining

The induction of apoptosis was determined with an Annexin V-PE Apoptosis Detection Kit I (BD Biosciences, Belgium). MCF-7 and MDA-MB-468 cells were seeded at 6 × 10^4^/well in 12-well plates. Cells were treated with ChPL with the indicated concentrations for 24 h and apoptosis was analyzed according to the manufacturer’s procedures. Following treatment with ChPL, cells were collected, washed with Annexin-binding buffer, and stained with Annexin V-phycoerythrin (PE) and 7-amino-actinomycin (7-AAD). Cells were further incubated at 15°C for 15 min in the dark and flow cytometry (BD FACSCalibur) was used for sample analysis.

### Caspase Activity Determination

Caspase activity was determined with the FLICA Apoptosis Detection Kit (Immunochemistry Technologies, USA) with the use of a caspase inhibitor FLICA (Fluorochrome Inhibitor of Caspases), a carboxyfluorescein-labeled fluoromethyl ketone peptide. Procedures were carried out according to the manufacturer’s instructions. Briefly, MCF-7 and MDA-MB-468 cells were seeded at 6 × 10^4^/well in 12-well plates. Cells were treated with ChPL (0–5 μM) for 12 h after which cells were collected and a buffer containing the FLICA caspase inhibitor was added. Cells were further incubated for 1 h at 37°C under 5% CO_2_ and then washed with washing buffer. Flow cytometry (BD FACSCalibur) was used to determine the fluorescence intensity of fluorescein. The increase in caspase activity was determined by the fluorescence intensity emitted from FLICA probes bound to the active caspases.

### Western Blot Analysis

MDA-MB-468 cells and MCF-7 were treated with the indicated concentrations of ChPL for 24 h, and Western blot analysis was performed according to the previously published procedure (Kawiak et al., 2012b). The preparation of cytosolic and mitochondrial fractions for cytochrome c release evaluation was performed as previously described (Kawiak et al., 2012b). The following specific primary antibodies were used: anti-β-actin (1:1,000) (Cell Signaling, Danvers, MA, USA), anti-Bcl-2, anti-Bak, anti-Bax, and anti-Mcl-1 (1:250) (Santa Cruz, Heidelberg, Germany), anti-ERK1/2, anti-MEK1/2, anti-p-ERK1/2, and anti-p-MEK1/2 (1:1,000) (Cell Signaling), anti-cytochrome c (1:5,000) (Abcam, UK), and anti-HSP60 (1:1,000) (Cell Signaling). Membranes were incubated with primary antibodies overnight at 4°C after which a 1-h incubation with HRP-conjugated secondary antibodies (1:2000) (Cell Signaling) was carried out. Protein levels were determined by chemiluminescence (ChemiDoc; Bio-Rad, Waltham, MA, USA) with a HRP substrate (Thermo Scientific, MA, USA).

### AlphaScreen Analysis

The inhibition of ERK phosphorylation was examined with the bead-based amplified luminescent *proximity* homogeneous *assay* (AlphaScreen). The assay was used, according to manufacturer’s instructions, to detect phosphorylated ERK (SureFire p-ERK 1/2, (Thr202/Tyr204), PerkinElmer, Rodgau, Germany). MCF-7 and MDA-MB-468 cells were seeded at 2 × 10^4^/well in 96-well plates. Cells were pre-treated with ChPL and/or AG1478 (1 μM) for 1 h followed by a stimulation with EGF (epidermal growth factor) (50 ng/ml) for 30 min in 5% CO_2_ at 37°C. Cells were lysed by the addition of 50 µl of SureFire lysis buffer (PerkinElmer) and plates were incubated at room temperature with agitation for 10 min (∼350 rpm). To determine p-ERK1/2 levels, cell lysates (4 µL) were transferred to a 384-well plate (Proxiplate, PerkinElmer) followed by the addition of the Reaction mix (7 µl) containing the Reaction buffer, Activation buffer, and Acceptor and Donor beads. Plates were incubated for 2 h at room temperature. The fluorescent signals were read with an Envision Multilabel Reader (PerkinElmer) with standard AlphaScreen settings.

### ERK1/2 Silencing

MDA-MB-468 and MCF-7 cells were transiently silenced with ERK1 and ERK2 siRNA or control, scrambled siRNA according to the manufacturer’s directions (Santa Cruz, Germany). The confirmation of ERK1/2 silencing was analyzed with Western blot, 24 h post-transfection. Apoptosis following ERK1/2 silencing was carried out with Annexin V-PE/7AAD staining after a 24-h transfection followed by a further 24-h treatment with ChPL. Mcl-1 expression was analyzed 24 h post-transfection with Western blot after the treatment of cells with ChPL for 24 h.

### Statistical Analysis

Values are expressed as means ± SEM of at least three independent experiments. Statistical analysis was performed with GraphPad Prism software by one-way ANOVA with Tukey’s *post hoc* tests. A *p* value of <0.05 was considered as statistically significant in each experiment.

## Results

### ChPL Inhibits the Proliferation of Breast Cancer Cells

**Figure 1:**
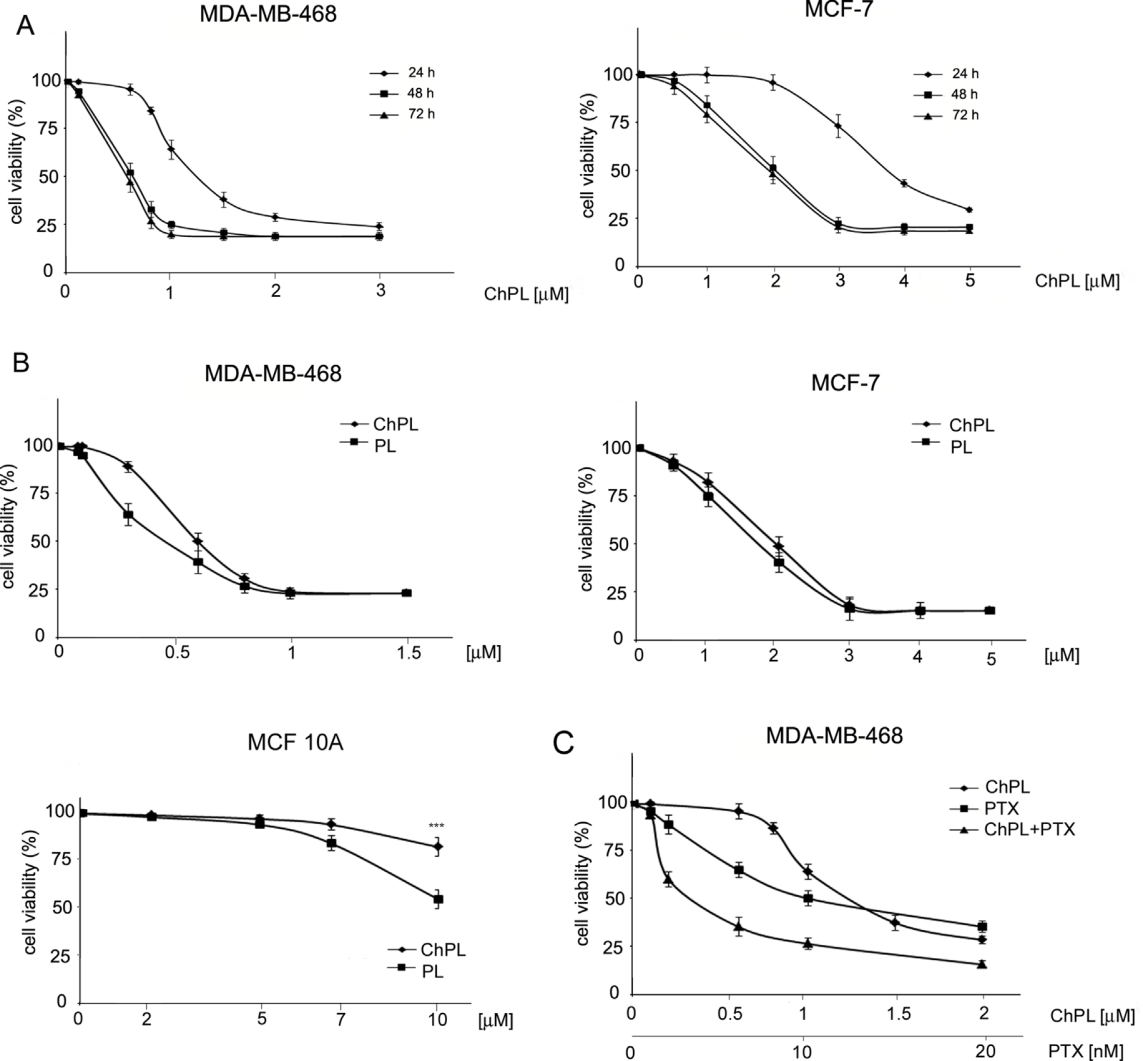
Effects of 3-chloroplumbagin (ChPL) and plumbagin (PL) on the viability of MDA-MB-468 and MCF-7 breast cancer cells and breast epithelial cells, MCF10A **(A)** Dose-dependent effects of ChPL on the reduction in cell viability. Cell survival was assessed with the MTT assay, n = 3. **(B)** Comparison in activity between ChPL and PL. Cell survival was assessed with the MTT assay, n = 3. Data were analyzed by one-way ANOVA with Tukey’s *post hoc* tests [*p* < 0.001 (***)]. **(C)** Combined effects of ChPL and paclitaxel (PTX) on the viability of MDA-MB-468 cells. Cell survival was assessed with the MTT assay, n = 3.

The effects of ChPL on MDA-MB-468 and MCF-7 breast cancer cell proliferation were determined with the use of the MTT assay over a period over 24 to 72 h. The IC_50_ value of ChPL after 24 h was 1.2 and 3.7 µM for MDA-MB-468 and MCF-7 cells, respectively. After a further 24 h of incubation, the IC_50_ values decreased to 0.6 and 2 µM, for MDA-MB-468 and MCF-7 cells, respectively. The IC_50_ values for ChPL did not decrease significantly with further incubation (**Figure 1A**). Further analysis was performed to compare the anti-proliferative activity of plumbagin and its 3-chloro-derviative. The effects of ChPL and PL on the proliferation of MDA-MB-468 and MCF-7 cells were analyzed after a 72-h incubation. Data analysis revealed that after this period, the IC_50_ value of plumbagin was comparable to that of ChPL (**Figure 1B**). The IC_50_ values for MDA-MB-468 cells were 0.6 and 0.4 µM for ChPL and PL, respectively, and 2 and 1.8 µM for ChPL and PL for MCF7 cells, respectively. Furthermore, the anti-proliferative activity of both compounds was analyzed toward a control, immortalized breast epithelial cell line, MCF 10A. ChPL displayed significantly lower cytotoxicity toward MCF 10A cells than PL. At the highest examined concentration of 10 µM, plumbagin decreased cell viability by 46% after a 72-h incubation period, whereas at this time point, the viability of ChPL-treated cells was 82% (**Figure 1B**).

To further evaluate the therapeutic potential of ChPL, the effects of ChPL on the activity of paclitaxel (PTX) toward breast cancer cells were evaluated. MDA-MB-468 cells, which were more sensitive to ChPL treatment, were treated with combination doses of ChPL and PTX in the dose range of 0.1 to 2 µM (ChPL) and 0.1 to 20 nM (PTX). As shown in **Figure 1C**, combination treatment with ChPL and PTX significantly reduced cell viability in comparison to single agent treatments. The combination index (CI) value obtained for MDA-MB-468 was 0.6, indicating a synergistic activity between ChPL and PTX.

### ChPL Induces Apoptosis and Inhibits Mcl-1 Expression in Breast Cancer Cells

**Figure 2:**
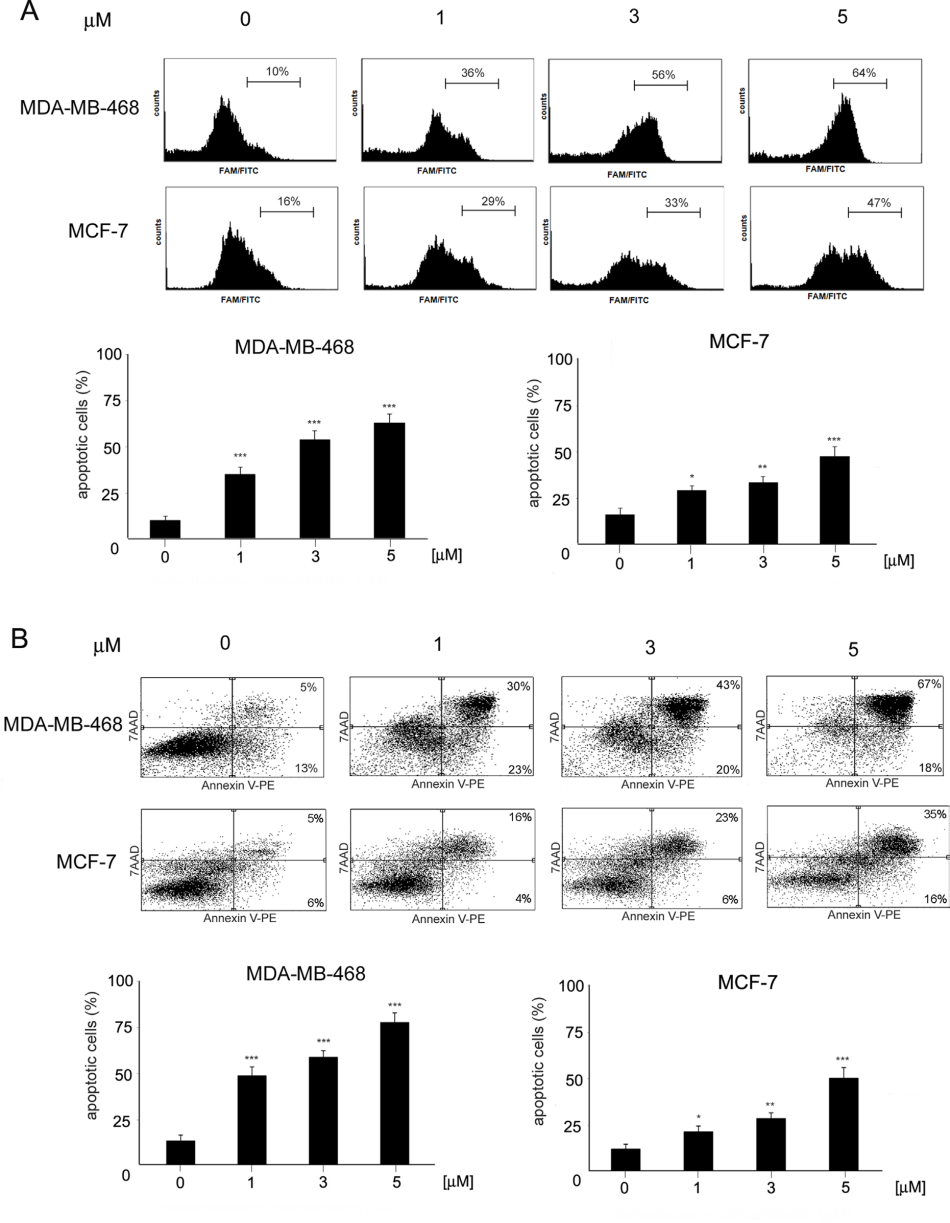
Induction of apoptosis by 3-chloroplumbagin in breast cancer cells. **(A)** Induction of caspase activity by ChPL. Analysis was performed with flow cytometry with the use of a caspase inhibitor, FAM-VAD-FMK **(B)** Apoptotic changes in plasma membrane induced by ChPL. Analysis was performed with flow cytometry after Annexin V-PE/7-AAD staining. Data were analyzed by one-way ANOVA with Tukey’s *post hoc* tests [*p* < 0.05 (*), *p* < 0.01 (**), *p* < 0.001 (***), n = 3].

To determine the mode of cell death induced by ChPL in breast cancer cells, characteristic features of apoptotic cell death were examined. Phosphatidylserine externalization and caspase induction were determined in ChPL-treated MDA-MB-468 and MCF-7 cells. Cytometric analysis of caspase activation revealed a dose-dependent increase in apoptosis induction in both cell lines after a 12-h treatment with ChPL. Similarly to the results of cell viability determination, the MDA-MB-468 cells were more sensitive to ChPL-induced cell death. At the concentration of 1 µM, a 25% increase in caspase activation was observed and at 5 µM, caspase induction increased by around 55%. In the case of MCF-7 cells, a 13% and 30% increase in caspase activity induction was observed at the ChPL concentration of 1 µM and 5 µM, respectively (**Figure 2A**). In accordance with caspase induction, ChPL also induced phosphatidylserine externalization in breast cancer cells, as analyzed by Annexin V staining. Cytometric analysis of MDA-MB-468 and MCF-7 cells treated with ChPL for a period of 24 h revealed cells mainly in the late-apoptotic stage. At the concentrations of 1 and 5 µM, the percentage of apoptotic cells increased by 30% and 60% in MDA-MB-468 cells, respectively, whereas in MCF-7 cells at these concentrations, a 10% and 40% increase in apoptotic cell populations were observed (**Figure 2B**).

**Figure 3:**
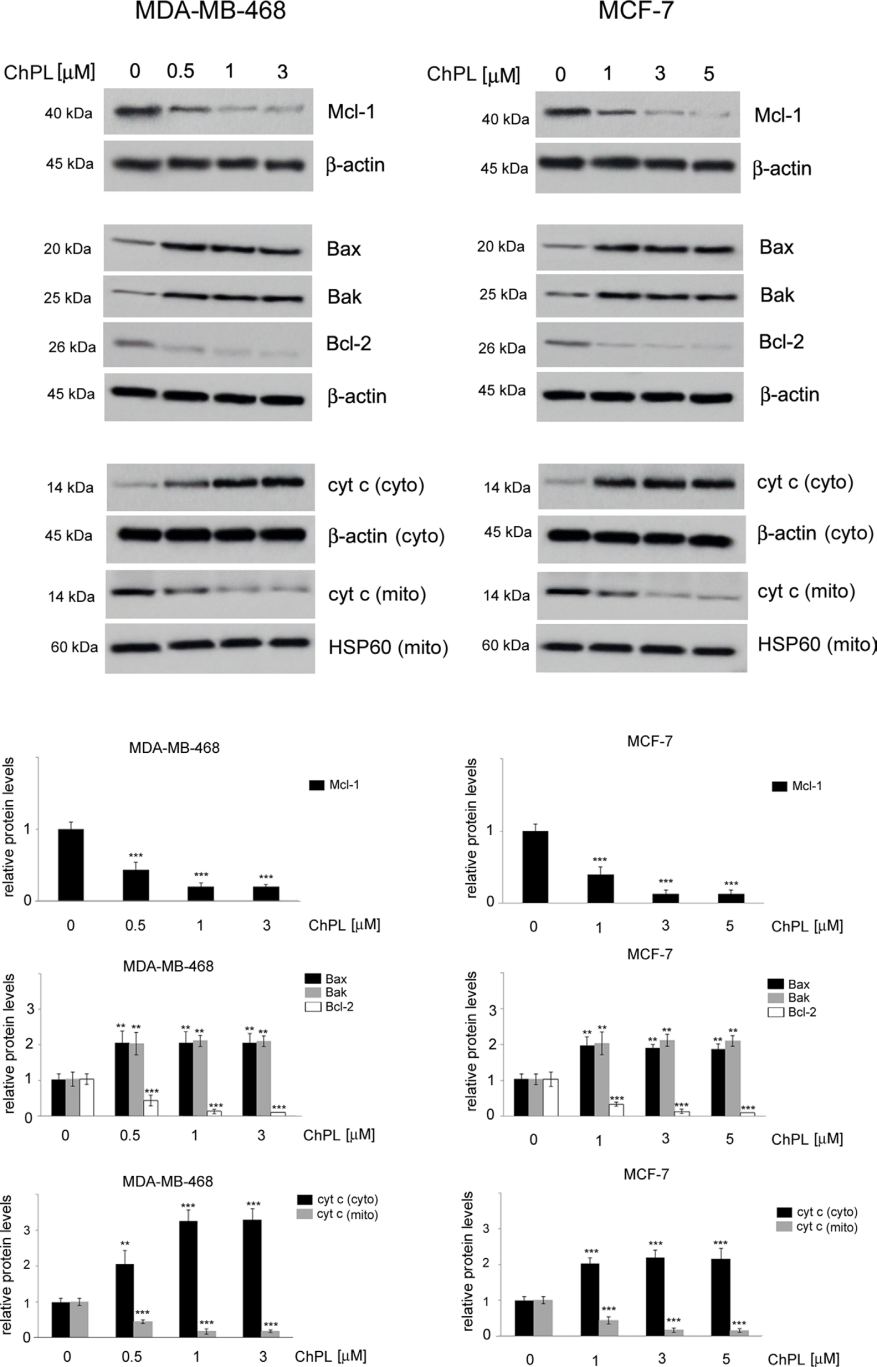
Effects of 3-chloroplumbagin on the expression levels of Bcl-2 family proteins and cytochrome c in breast cancer cells. MDA-MB-468 and MCF-7 cells were treated with the indicated concentrations of ChPL for 24 h, and the levels of Mcl-1, Bax, Bak, Bcl-2 and cytochrome c were assessed by Western blot analysis. Densitometric analysis represents protein levels normalized to β-actin levels or HSP60 levels. Data were analyzed by one-way ANOVA with Tukey’s *post hoc* tests [*p* < 0.01 (**), *p* < 0.001 (***), n = 3].

Since naphthoquinones have previously been reported to induce apoptosis through the mitochondria-mediated pathway, the involvement of the Bcl-2 family proteins in ChPL-induced cell death was further examined. MDA-MB-468 and MCF-7 cells were treated for 24 h with ChPL, and the levels of pro-apoptotic and anti-apoptotic Bcl-2 proteins were determined with Western blot analysis. The results showed that the levels of anti-apoptotic Bcl-2 and Mcl-1 decreased upon ChPL treatment in both cell lines. A significant decrease in Bcl-2 and Mcl-1 levels was observed in MDA-MB-468 and MCF-7 cells at the lowest concentrations tested. Furthermore, ChPL increased the levels of pro-apoptotic proteins, Bax and Bak, in MDA-MB-468 and MCF-7 cells. In accordance with the results of the anti-apoptotic proteins, the lowest examined concentrations of ChPL significantly increased pro-apoptotic protein levels (**Figure 3**, **Supplementary Figure 1**). The activation of the pro-apoptotic Bcl-2 proteins leads to the permeabilization of the mitochondria outer membrane and the subsequent release of cytochrome *c*; thus, the effects of ChPL on cytochrome c release were examined. Western blot analysis revealed an increase in cytochrome c levels in the cytosol of MDA-MB-468 and MCF-7 cells upon treatment with ChPL. Cytochrome c increase in the cytosol was accompanied by a decrease of cytochrome c in the mitochondrial fraction of cells (**Figure 3**, **Supplementary Figure 2**).Taken together, these data indicate that ChPL induces apoptosis through the mitochondria-mediated pathway.

### ChPL** Inhibits MAPK Signaling in Breast Cancer Cells**

**Figure 4:**
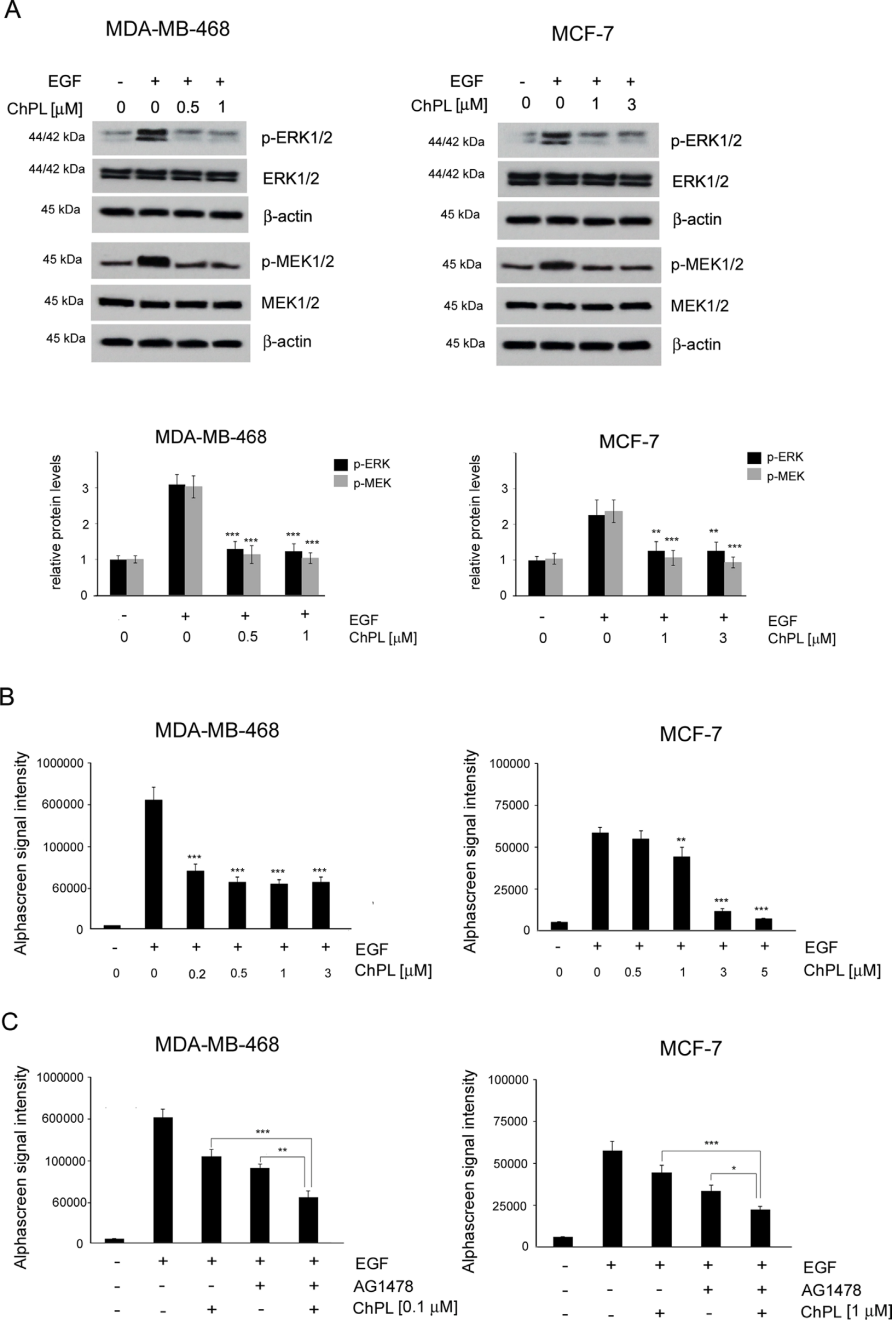
Effects of 3-chloroplumbagin on the inhibition of MAP kinase signaling in breast cancer cells. **(A)** Effects of ChPL on the expression levels of proteins involved in MAP kinase signaling. Protein levels were assessed by Western blot analysis. Densitometric analysis represents protein levels normalized to total ERK1/2 and MEK1/2 levels. **(B)** Effects of ChPL on the levels of p-ERK 1/2 (Thr202/Tyr204) in MDA-MB-468 and MCF-7 cells. Protein levels were determined with the AlphaScreen assay. **(C)** Effects of ChPL and AG1478 on the levels of p-ERK 1/2 (Thr202/Tyr204) in MDA-MB-468 and MCF-7 cells. Protein levels were determined with the AlphaScreen assay. Data were analyzed by one-way ANOVA with Tukey’s *post hoc* tests [*p* < 0.05 (*), *p* < 0.01 (**), *p* < 0.001 (***); n = 3].

To determine the effects of ChPL on MAPK signaling in breast cancer cells, MDA-MB-468 and MCF-7 cells were treated with ChPL and Western blot analysis was performed to examine the effects of ChPL on levels of phosphorylated MEK and ERK in breast cancer cells. Cells were pretreated for 24 h with plumbagin after which the levels of p-ERK and p-MEK were stimulated with EGF for 30 min. The results of Western blot analysis revealed that ChPL reduces the levels of phosphorylated MEK and ERK, induced by EGF (**Figure 4A**, **Supplementary Figure 3 and 4**). The results obtained with Western blot analysis were confirmed with quantitative AlphaScreen assay analysis of the influence of ChPL on p-ERK inhibition. The AlphaScreen assay is a proximity-based assay employing streptavidin-coated beads conjugated with a biotinylated peptide substrate and acceptor beads with an anti-phosphotyrosine antibody. Provided that the phosphorylation of the ERK protein occurs, the beads are brought into proximity through the binding of the phosphospecific antibody on the acceptor bead. The excitation of donor beads induces the release of singlet oxygen molecules that triggers a cascade of energy transfer in the acceptor beads that results in light emission. To determine the effects of ChPL on p-ERK inhibition, cells were pretreated for 1 h with ChPL after which p-ERK was induced with EGF for 30 min. As demonstrated in **Figure 4B**, the relative levels of endogenous phosphorylated ERK at residues Thr202 and Tyr 204 were induced with EGF stimulation in MDA-MB-468 and MCF-7 cells. The treatment of cells with ChPL markedly reduced the levels of EGF-induced p-ERK, and at the concentrations of 0.2 µM and 1 µM a significant reduction in the levels p-ERK were detected in MDA-MB-468 and MCF-7 cells, respectively (**Figure 4B**). Further experiments were conducted to evaluate the ability of ChPL to synergistically interact with an EGF inhibitor (AG1478) to inhibit MAPK/ERK signaling in breast cancer cells. Cells were pretreated for 1 h with ChPL and/or AG1478, after which p-ERK was induced with EGF for 30 min. The results of the Alpha Screen assay showed a significant reduction in p-ERK in cells pre-treated with a combination of ChPL and AG1478 in comparison to cells treated with single agents (**Figure 4C**). Collectively, these results point to the ability of ChPL to inhibit MAP kinase signaling in breast cancer cells.

### ChPL Induces Apoptosis in Breast Cancer Cells Through MAPK-Mediated Mcl-1 Inhibition

**Figure 5:**
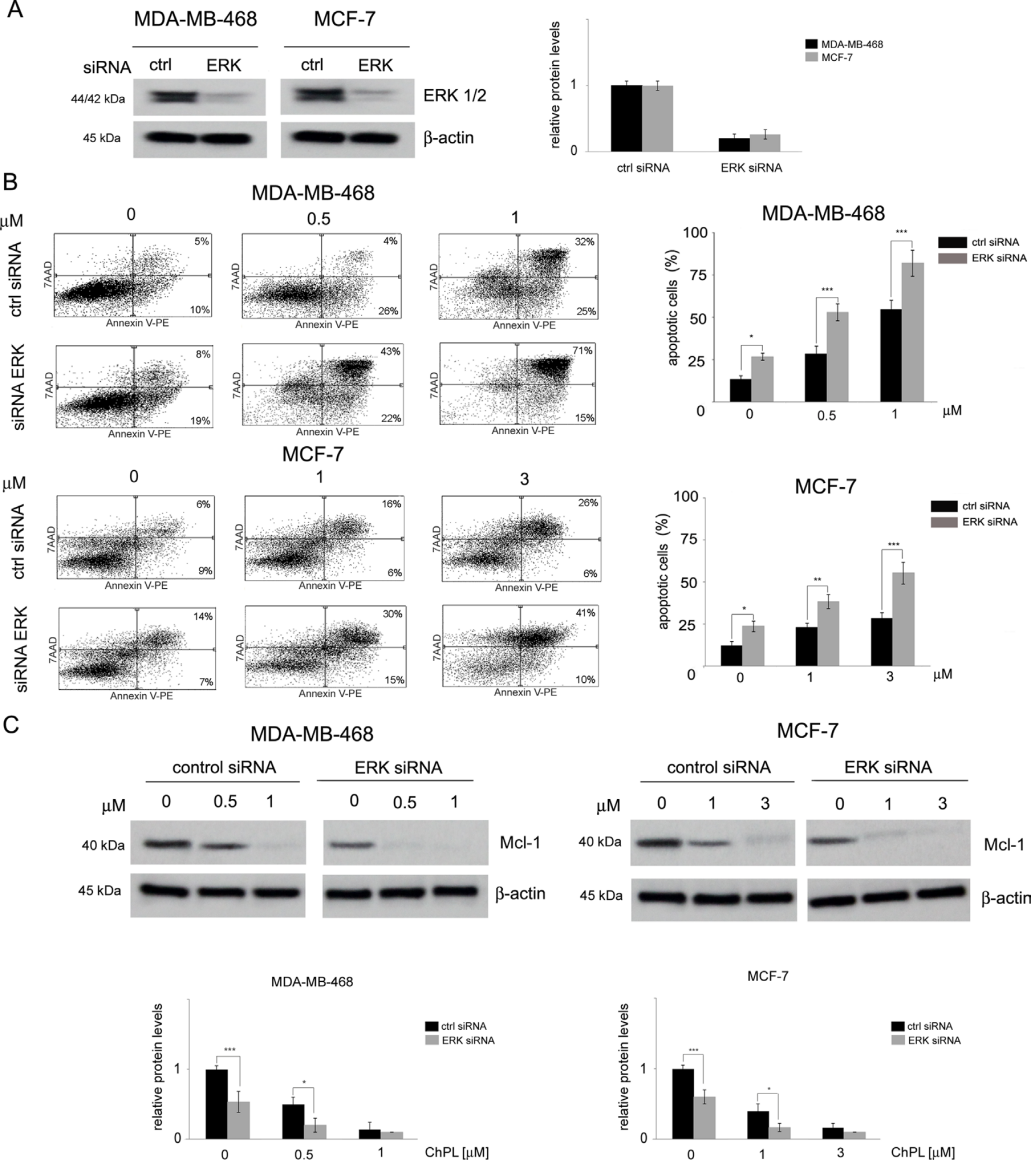
The role of MAPK signaling in 3-chloroplumbagin-mediated ERK inhibition and apoptosis induction. **(A)** Silencing of ERK1/2 with ERK1/2 siRNA and control siRNA in MDA-MB-468 and MCF-7 cells. Protein levels were determined with Western blot analysis. Densitometric analysis represents ERK1/2 levels normalized to β–actin levels. **(B)** Apoptosis induction by ChPL in MDA-MB-468 and MCF-7 cells transfected with ERK siRNA or control siRNA. Apoptosis induction was assessed with flow cytometry after Annexin V-PE staining. **(C)** Influence of ERK silencing on expression levels of Mcl-1 in MDA-MB-468 and MCF-7 cells treated with ChPL. Mcl-1 levels were assessed with Western blot analysis. Densitometric analysis represents Mcl-1 levels normalized to β-actin levels. Data were analyzed by one-way ANOVA with Tukey’s *post hoc* tests [*p* < 0.05 (*), *p* < 0.01 (**), *p* < 0.001 (***), n = 3].

The involvement of MAPK signaling inhibition in ChPL-mediated apoptosis induction was examined by silencing the expression of ERK in MDA-MB-468 and MCF-7 cells and determining the potential of ChPL to induce apoptosis in these cells. Cells were transiently transfected with ERK siRNA or control, scrambled siRNA and 24 h following transfection, Western blot analysis was performed to determine ERK levels and verify transfection efficacy. The results of Western blot analysis revealed decreased levels of ERK in MCF-7 and MDA-MB-468 cells transfected with ERK siRNA in comparison with cells transfected with control siRNA (**Figure 5A**, **Supplementary Figure 5**). To determine the role of ERK in ChPL-mediated apoptosis induction, 24 h post-transfection breast cancer cells were treated with ChPL for 24 h and apoptosis induction was assessed with Annexin V-PE staining. In cells with reduced ERK expression levels, an increase in the percentage of apoptotic was observed in both cell lines. Furthermore, cells with silenced ERK expression were significantly more sensitive to treatment with ChPL in comparison with control cells, transfected with scrambled siRNA (**Figure 5B**). The role of ERK in ChPL-mediated Mcl-1 inhibition was further examined. Similarly to the results obtained with Annexin V staining, Western blot analysis of Mcl-1 expression levels revealed a reduction of Mcl-1 in cells with silenced ERK expression. The treatment of cells with ChPL reduced Mcl-1 levels to a greater extent in cells transfected with ERK siRNA than in control cells (**Figure 5C**, **Supplementary Figure 6**), indicating the involvement of ERK in ChPL-mediated Mcl-1 inhibition.

## Discussion

Mcl-1 is an anti-apoptotic protein of the Bcl-2 protein family, which participates in regulating the intrinsic apoptotic pathway (IAP) (Han et al., 2004). The intrinsic pathway is characterized by mitochondria outer membrane permeabilization (MOMP) which results in the release of cytochrome c from the mitochondria into the cytosol where it takes part in caspase activation. The MOMP is regulated by Bcl-2 family effectors (Bax and Bak) which heterodimerize creating pores in the outer mitochondria membrane. Bak is associated with the outer mitochondrial membrane whereas Bax transfers from the cytosol to the mitochondria in response to apoptotic stimuli. The ability of Bax and Bak to interact is determined by conformational changes induced through interactions with pro-apoptotic, BH3-only Bcl-2 proteins (Bad, Bid, Bim, Puma, Noxa). MOMP is prevented by anti-apoptotic Bcl-2 proteins comprising two groups, one consisting of Bcl-2, Bcl-xL, Bcl-w, and the other of Mcl-1 and Bcl2A1, which bind pro-apoptotic Bcl-2 proteins preventing MOMP. Thus, the ratio of apoptotic to anti-apoptotic proteins is critical in regulating cellular susceptibility to apoptosis (Cory and Adams, 2002; Adams and Cory, 2007; Youle and Strasser, 2008).

Bcl-2 family protein expression is regulated through various signaling pathways, including the MAP kinase pathway. MAP kinase signaling plays a significant role in breast cancer progression (Mueller et al., 2000). The activation of the MAP kinase pathway has been shown to suppress apoptosis induction through the regulation of anti-apoptotic and pro-apoptotic Bcl-2 family protein activity (Boucher et al., 2000). ERK activation has been demonstrated to inactivate the pro-apoptotic protein BAD through the phosphorylation at Ser112 by ERK-activated p90 ribosomal S6 kinase (RSK) (Bonni et al., 1999). ERK has also been shown to increase cell survival through enhancing the stability and anti-apoptotic activity of Mcl-1 through phosphorylation of Mcl-1 at Thr163 (Domina et al., 2004). Research has shown that MAP kinase signaling is involved in the regulation of Mcl-1 transcription (Wang et al., 2003). The induction of MAPK signaling through EGF stimulation up-regulated Mcl-1 in breast cancer cells. On the other hand, the use of a MAP kinase inhibitor U0126 protected breast cancer cells from EGF-induced Mcl-1 overexpression (Booy et al., 2011). Similarly, the present research showed that the knockdown of ERK in breast cancer cells decreased Mcl-1 expression and increased the induction of apoptosis in breast cancer cells. Furthermore, the downregulation of ERK expression increased the sensitivity of breast cancer cells to ChPL-induced apoptosis induction. Accordingly, ChPL was shown to inhibit MAP kinase signaling and downregulate Mcl-1 expression. The downregulation of Mcl-1 by ChPL was associated with MAPK inhibition as shown by the enhanced ChPL-induced Mcl-1 downregulation in cells with silenced ERK.

The anti-apoptotic Bcl-2 proteins are well-recognized drug targets and several Bcl-2 protein inhibitors have been synthesized and some have entered clinical trials (Doi et al., 2012; Hamdy et al., 2013). Most inhibitor design studies have mainly focused on the Bcl-2 and Bcl-xL proteins. However, several studies have shown the Mcl-1 protein to be an important target in drug design. The most effective Bcl-2 inhibitors ABT-737 and its analog ABT-263 target Bcl-2 and Bcl-xL (Oltersdorf et al., 2005; Yip and Reed, 2008); however, only weakly inhibited Mcl-1 thus are not effective against cancers with elevated Mcl-1 levels (van Delft et al., 2006). Furthermore, resistance acquired toward ABT-737 and ABT-263 has been shown to be associated with the up-regulation of Mcl-1 (Lin et al., 2007; Yecies et al., 2010; Wang et al., 2014). The downregulation of Mcl-1 sensitized cells to ABT-737, thus showing that combined therapy with Mcl-1 inhibitors could increase therapy efficacy (Konopleva et al., 2006; Chen et al., 2007).

In this study, the ability of ChPL to induce apoptosis in breast cancer cells through the mitochondria-mediated intrinsic pathway was shown. Apoptosis induction by ChPL was associated with the downregulation of anti-apoptotic Bcl-2 and Mcl-1. The compensatory nature of anti-apoptotic Bcl-2 family proteins points to the importance of targeting both subgroups of the anti-apoptotic Bcl-2 family proteins to achieve long-term treatment efficacy (Vogler et al., 2009). The importance of targeting Mcl-1 in breast cancers is supported by studies carried out on Bcl-2 family proteins examining their upregulation in various types of breast cancers. Mcl-1 upregulation has been determined in 28% of human breast cancer cell lines as reported by the cancer cell line encyclopedia (CCLE), whereas Bcl-2 is upregulated in 3% (Williams and Cook, 2015). In triple negative breast cancers following neoadjuvant chemotherapy, *MCL1* expression was amplified in 54% of the examined samples (Balko et al., 2014). Furthermore, irrespective of tumor type, Mcl-1 upregulation has been associated with high tumor grade and a decrease in patient survival (Ding et al., 2007). Research showing that Mcl-1 upregulation significantly contributes to resistance against widely used anticancer therapies including antitubulins (Wertz et al., 2011), HER2-targeting agents (Bashari et al., 2016), points to Mcl-1 as an important target in breast cancer therapy.

**Figure 6:**
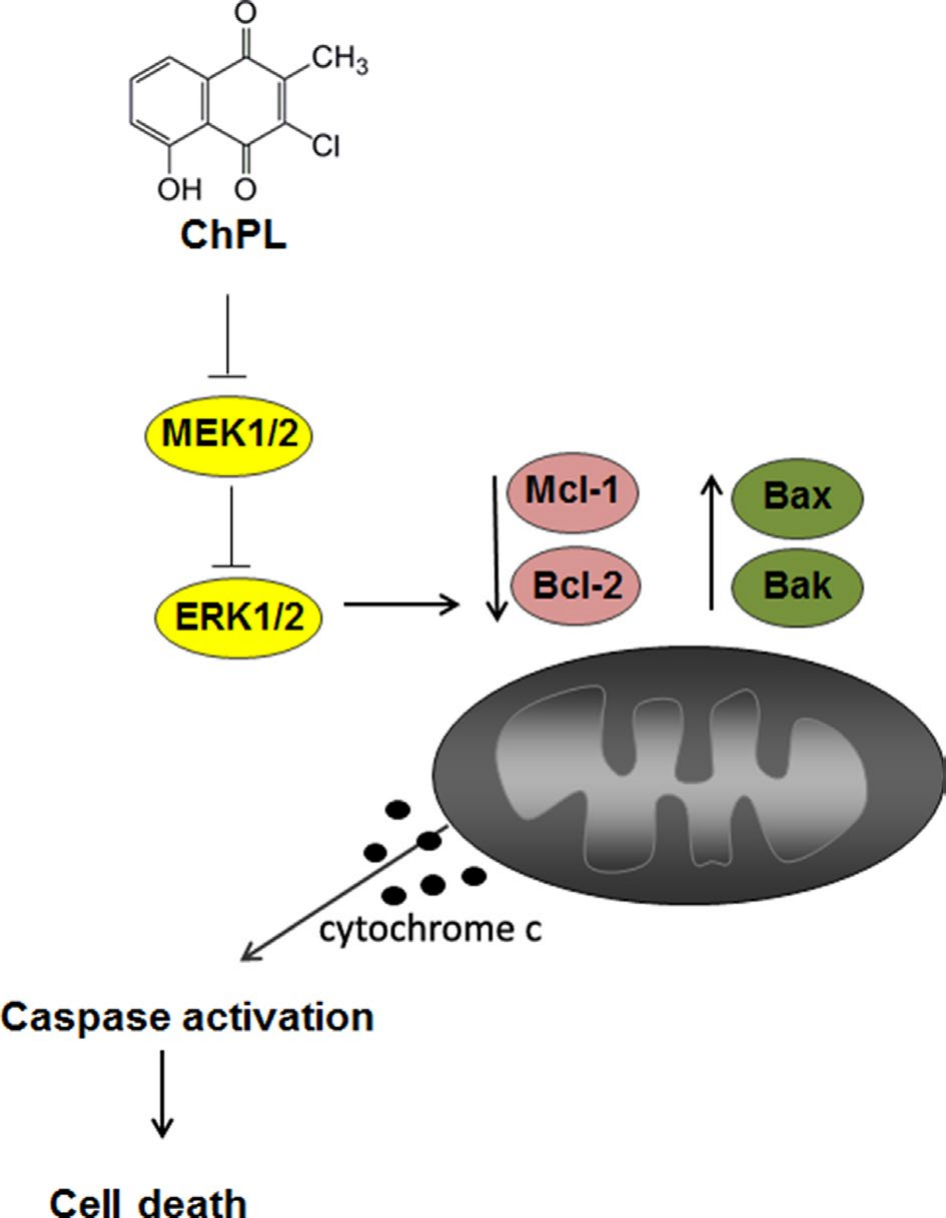
The proposed mechanism of apoptosis induction by 3-cloroplumbagin (ChPL) in breast cancer cells. ChPL inhibits MAP kinase signaling and induces apoptosis through the activation of the mitochondria-mediated pathway, involving the downregulation of anti-apoptotic proteins Mcl-1 and Bcl-2 and upregulation of pro-apoptotic Bcl-2 family proteins.

The results of the research presented herein show that ChPL induces apoptosis in breast cancer cells through MAP kinase signaling inhibition and activation of the mitochondria-mediated pathway involving Mcl-1 downregulation (**Figure 6**). The activity of ChPL was comparable to that of its precursor, plumbagin. PL is an extensively researched naphthoquinone with promising anti-cancer potential. Plumbagin has been reported to selectively inhibit the proliferation of breast cancer cells and suppress breast tumor xenograft growth in mice (Kuo et al., 2006). In the present research, ChPL displayed similar activity toward breast cancer cells as plumbagin. The therapeutic potential of ChPL can be emphasized by the lower cytotoxicity of ChPL toward non-tumorigenic, epithelial MCF 10A cells in comparison to plumbagin. Furthermore, in the presented research, ChPL displayed higher activity toward triple negative breast cancer cells, MDA-MB-468, which is the most aggressive breast cancer subtype. Since the expression of *MCL-1* in triple negative breast cancer is high, the ability of ChPL to decrease Mcl-1 levels in MDA-MB-468 cells points to its promising potential in breast cancer treatment. Moreover, ChPL inhibited both the Mcl-1 and Bcl-2 arm of the anti-apoptotic Bcl-2 family proteins, which is important in minimizing drug resistance occurrence. These findings encourage further investigation into the anticancer potential of ChPL, specifically toward breast cancer cells.

## Data Availability

All datasets generated for this study are included in the manuscript and the supplementary files.

## Author Contributions

AKa conceived, designed and performed experiments, interpreted data, wrote the manuscript, and acquired funding. AD, AKr, and MS performed the experiments and the data analysis. AKr and EL revised the article.

## Funding

This work was supported by the grant of the National Science Centre No. 2011/03/B/NZ7/06144. Open access publication supported by the Polish Ministry of Science and Higher Education [grant number DS: 530-M031-D754-18] and [grant number DS 530-M035-D673-19]

## Conflict of Interest Statement

The authors declare that the research was conducted in the absence of any commercial or financial relationships that could be construed as a potential conflict of interest.

## Abbreviations

7-AAD, 7-amino-actinomycin; CCLE, cancer cell line encyclopedia; ChPL, 3-chloroplumbagin; EGF, epidermal growth factor ERK, extracellular signal-regulated kinase; FLICA, Fluorochrome Inhibitor of Caspases; IAP, intrinsic apoptotic pathway; MOMP, mitochondria outer membrane permeabilization; MAPK, mitogen-activated protein kinase; MTT, (3-(4,5-dimethylthiazol-2-yl)-2,5-diphenyltetrazolium bromide; PL, plumbagin; PE, phycoerythrin; PTX, paclitaxel; RSK, ribosomal S6 kinase.
