# Supplementary material for: 3-Chloroplumbagin Induces Cell Death in Breast Cancer Cells Through MAPK-Mediated Mcl-1 Inhibition
Source: Front Pharmacol. 2019 Jul 26;10:784. doi: 10.3389/fphar.2019.00784 (PMC6675870; doi:10.3389/fphar.2019.00784)

Supplementary Figure 1: uncropped images of Western blots present in Figure 3

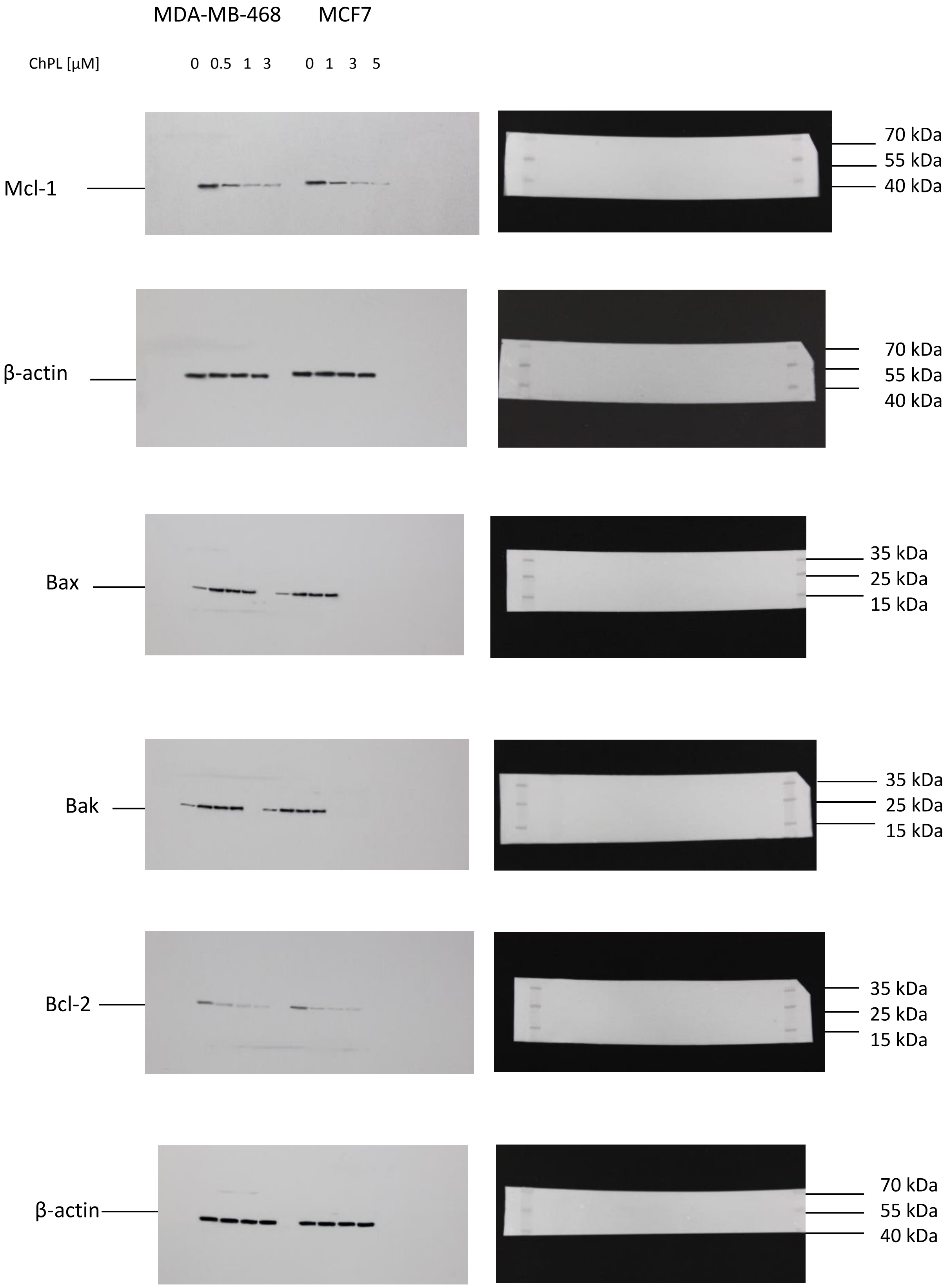

Supplementary Figure 2: uncropped images of Western blots present in Figure 3

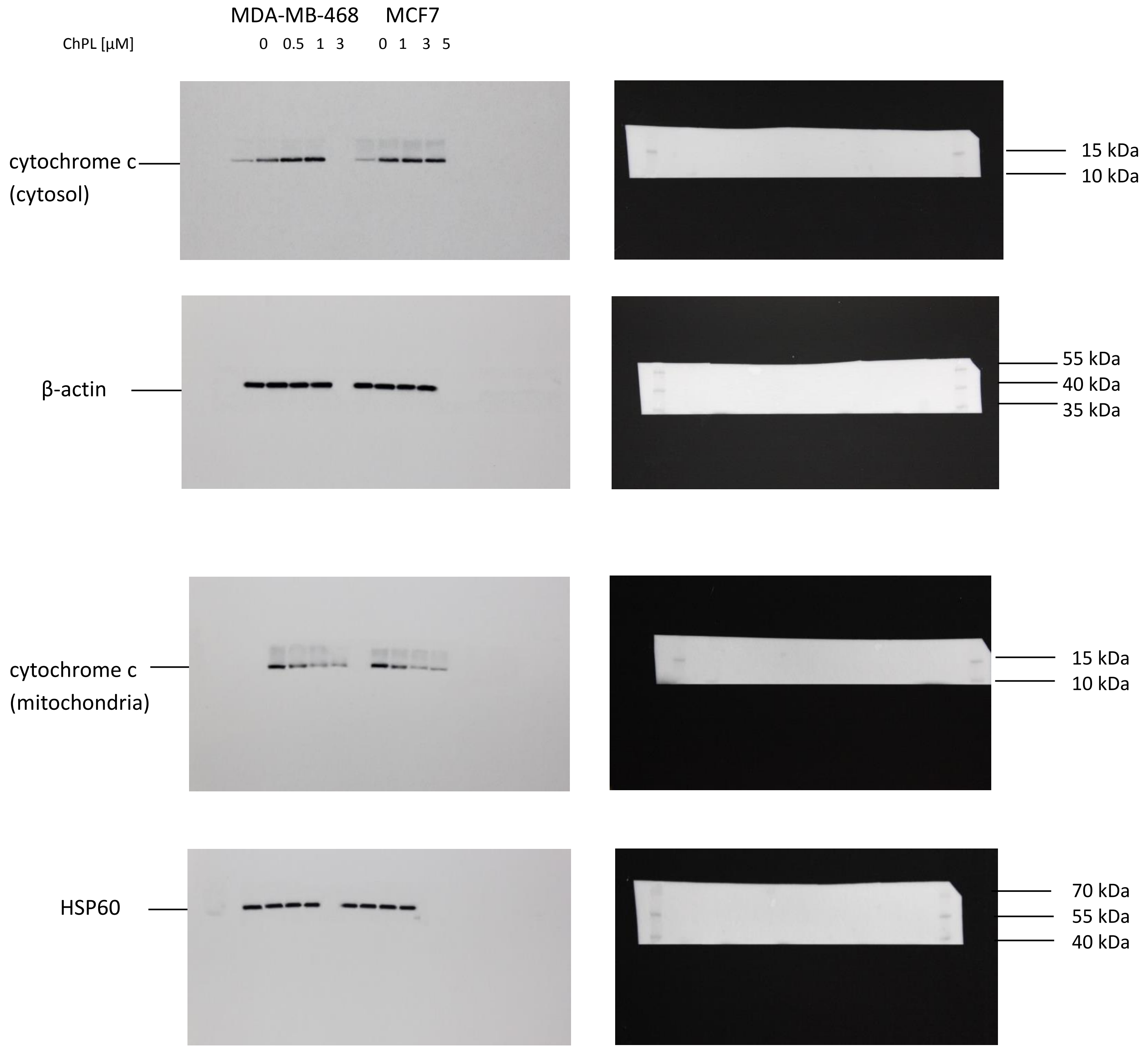

Supplementary Figure 3: uncropped images of Western blots present in Figure 4A

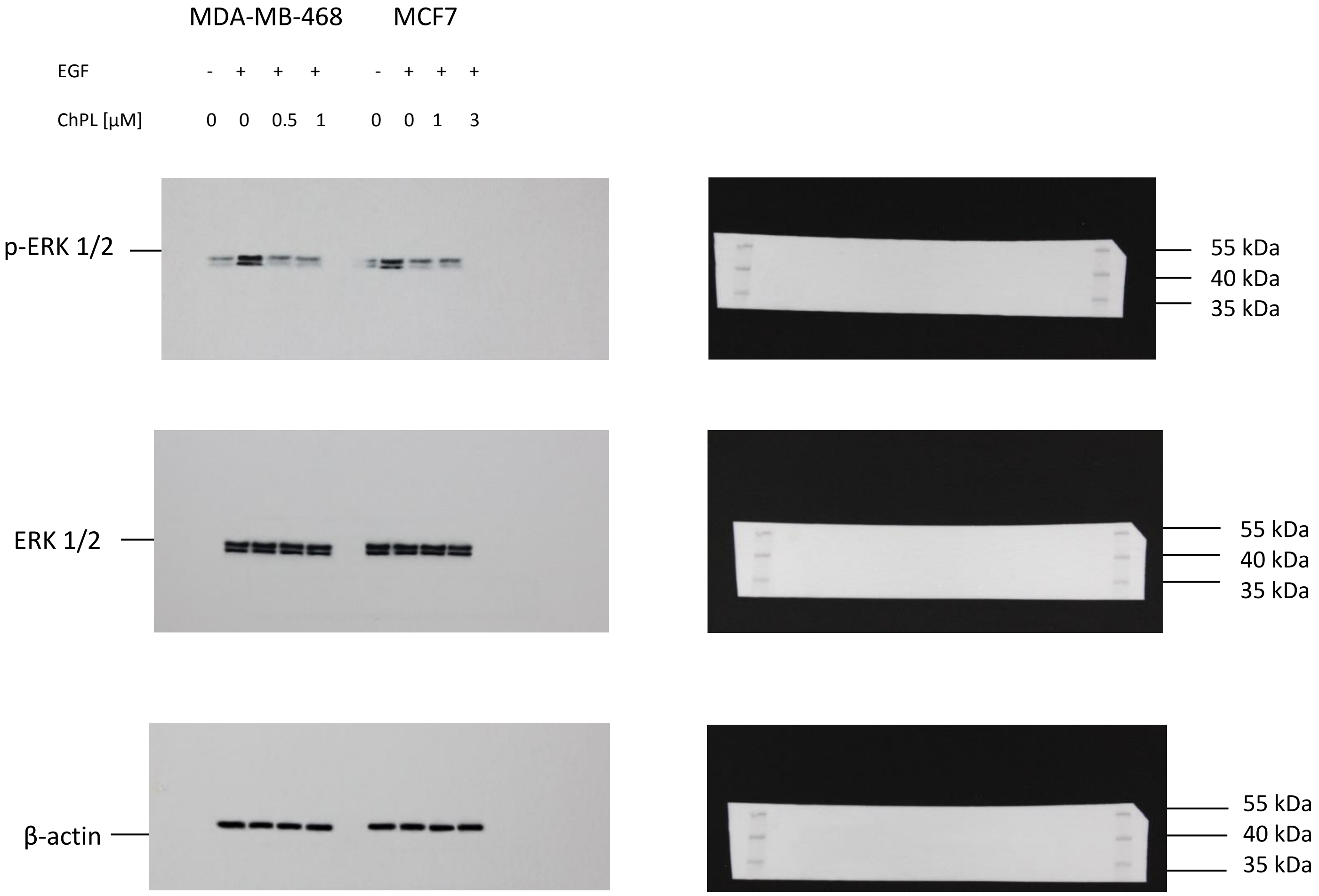

Supplementary Figure 4: uncropped images of Western blots present in Figure 4A

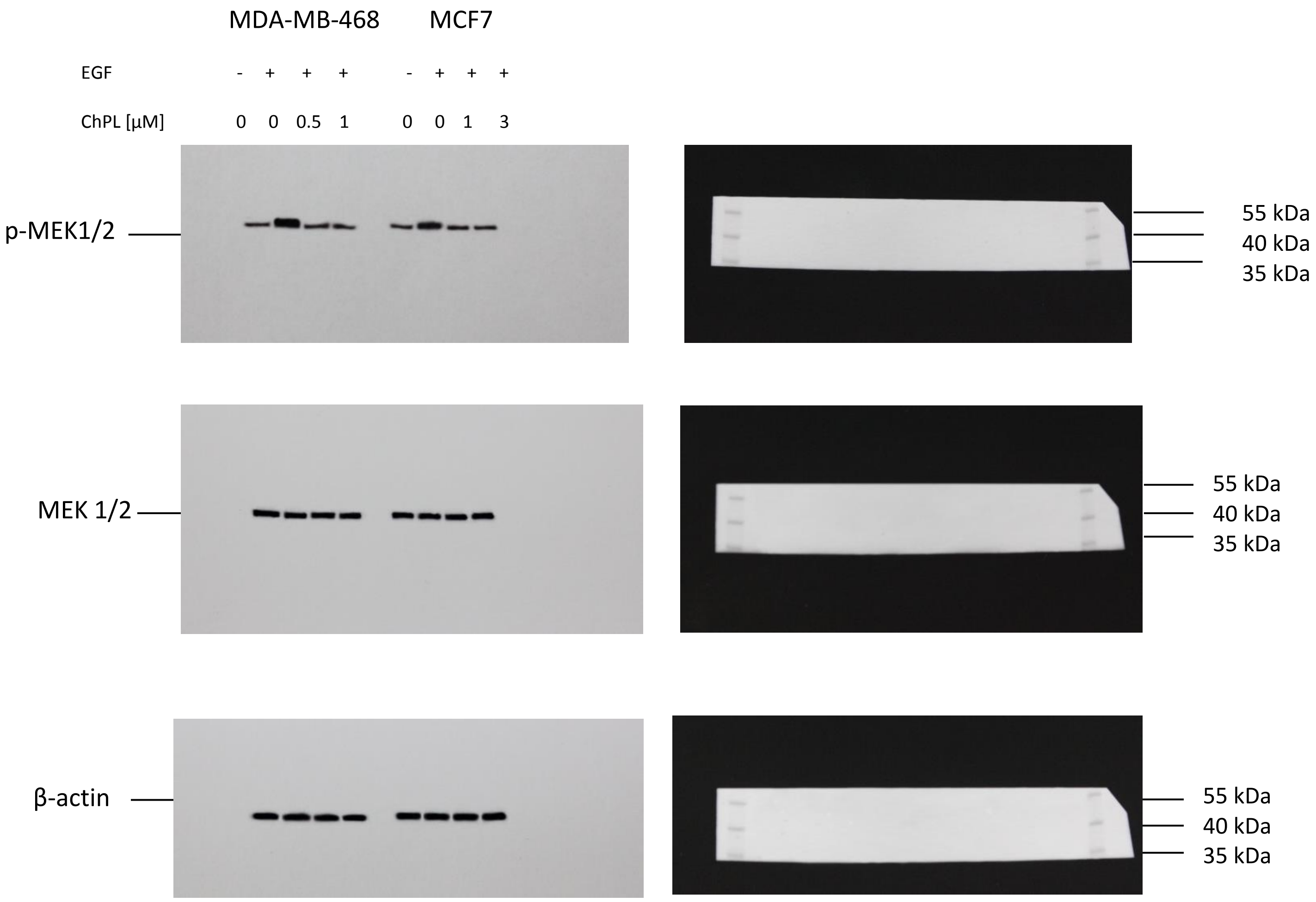

Supplementary Figure 5: uncropped images of Western blots present in Figure 5A

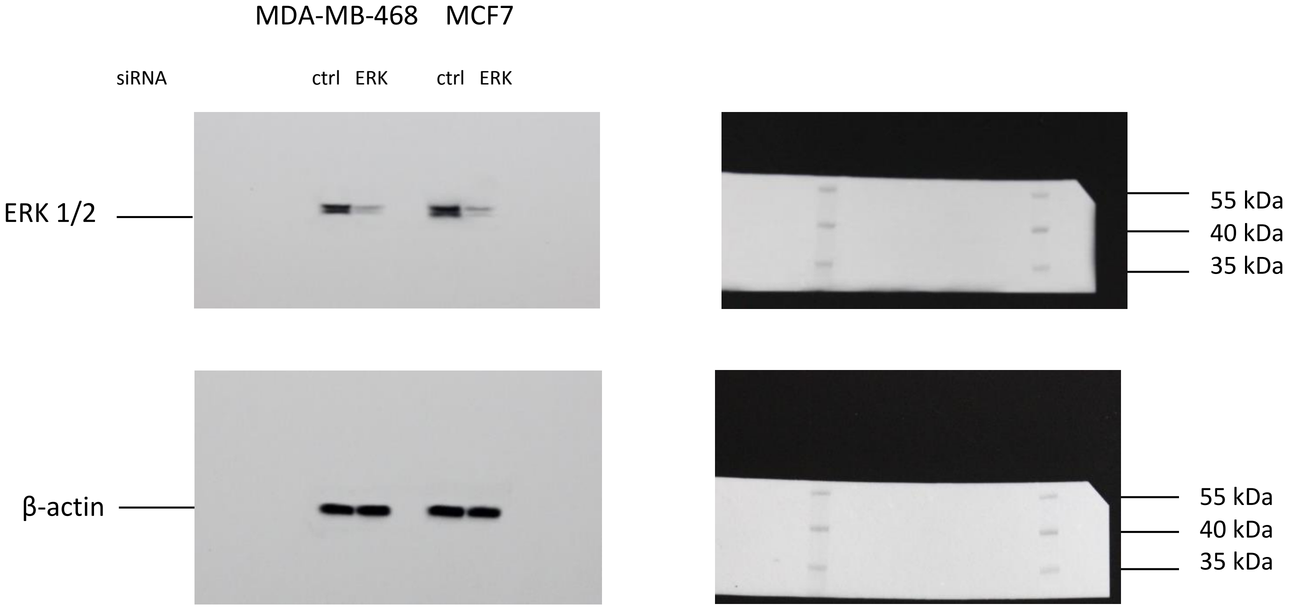

Supplementary Figure 6: uncropped images of Western blots present in Figure 5C

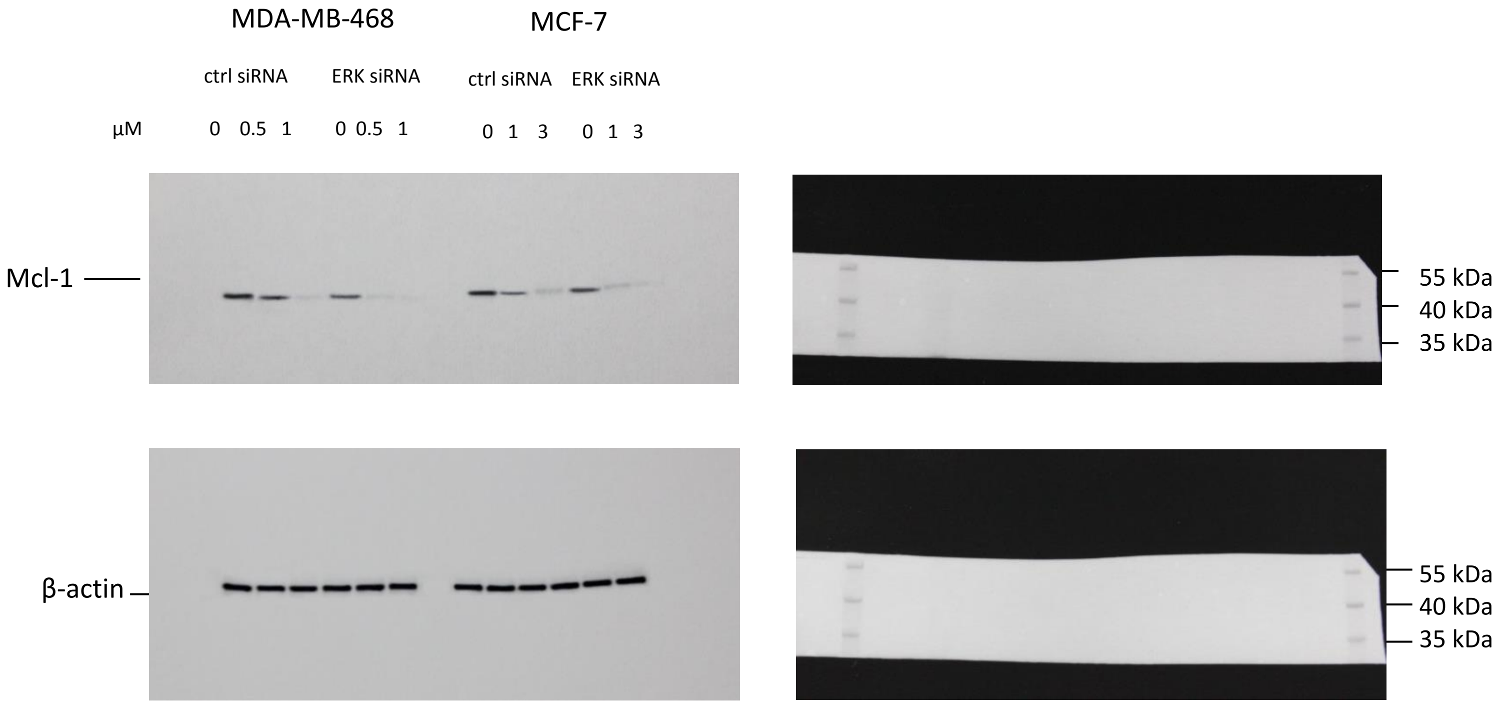

Supplement: Supplementary file 1 [file DataSheet_1.pdf]
